# Knowledge of Hazards Associated With Urban Livestock Farming in Southeast Nigeria

**DOI:** 10.3389/fvets.2021.600299

**Published:** 2021-03-02

**Authors:** Anthonia N. Asadu, Jane M. Chah, Clement O. Attamah, Edwin M. Igbokwe

**Affiliations:** Department of Agricultural Extension, University of Nigeria, Nsukka, Nigeria

**Keywords:** knowledge, livestock keeping, urban areas, farmers, Nigeria

## Abstract

The study assessed urban farmers' knowledge of health hazards associated with Urban Livestock Farming (ULF) in Southeast Nigeria. Multistage and random sampling techniques were used to select 210 respondents. Structured interview schedule was used to collect data. Data collected were analyzed using descriptive and inferential statistics. Major type of animals reared was broiler. A good proportion of the respondents had a high knowledge of hazards associated with livestock keeping. However, some farmers did not know that animal products from intensive system can be contaminated with heavy metals and that animal dung should be treated before use for crop cultivation. There was a significant influence of socio-economic characteristics of farmers on knowledge of hazards posed by livestock keeping. Farmers' knowledge of hazards varied significantly between the three states studied. Farmers therefore need technical advice to fully understand the hazards associated with urban livestock farming and their consequences as well as on pre-treatment of dung to reduce transfer of pathogens.

## Introduction

Globally, the growth of cities and urbanized areas continues at an exponential rate. The urban population of the world has grown from 751 thousand in 1950 to 4.2 million in 2018 and it is estimated that the world's population could add up to 2.5 billion people to urban areas by 2050, the highest urban growth rates being in developing countries (1). Projection shows that by 2060, most of Africa's inhabitants will live in urban areas as against 40% in 2010. This will increase to 50% by 2030 and 65% by 2060 making most of the urban centers megacities as in Asia and Latin America (2). Sub-Saharan Africa is the fastest urbanizing region in the world, with an urban population growth rate of 4.1% per annum, compared to 2% growth rate globally (3). About 40% of sub-Saharan African total population lives in urban areas and cities currently (4). As a result, the growth in urban poverty is rapidly outstripping that of rural poverty (5). Consequently, there is increasing concern about feeding the growing number of urban poor, many of whom have no permanent employment and limited access to resources.

Statistics show that by 2040, Nigeria's population growth would quadruple without commensurate amenities and employment and between 2018 and 2050 projections indicate that urban expansion will rise by 35% (6). As a result, the urban population of Southeast Zone of Nigeria is increasing alongside other urban centers in the country. The rate of rural-urban drift is greatly accelerated leading to urban expansion in southeast Nigeria. This condition poses great sustainable food security challenges for Nigerian urban centers.

About 40 million people in Nigeria are believed to be hungry and a large percentage of the population lacks access to adequate food (7). High inflation rate, food price instability and relatively low wages have made the average Nigerian liable to food insecurity (8). To survive, urban dwellers engage in urban farming (UF). Urban farming can be considered an integral part of viable strategies for sustainable urban development. Urban farming can be widely defined as any farming activity within the administrative boundary of an urban center (9, 10). It involves both growing of crops and animal husbandry within the city areas. Urban livestock farming (ULF) can contribute to some key challenges encountered by urban areas. As a result, some governments have reached a conclusion that for ULF to achieve its full potentials, it has to be controlled in order to reduce the associated risk (11).

The health hazards of ULF are probably the most significant fears that occupy the minds of development and urban planning professionals (12). Urban planners tend to believe that urban production presents a health risk because of specific use of wastewater in production systems. Standing water in irrigation channels is perceived as providing breeding grounds for mosquito which is an important vector in the transmission of malaria (13, 14). The perception and beliefs around the use of wastewater from urban ditches and streams represents a significant health issue. Livestock keeping can be harmful to urban environment. Free wandering animals can injure people, cause traffic accidents and destroy gardens (15). Animals kept in intensive system may be contaminated with pesticides. Animal dung left to decompose on compounds or by road sides could act as sources of harmful bacteria to humans and other animals.

Center for Disease Control and Prevention (16) indicated that agriculture is one of the most hazardous sectors in the world. Agricultural workers suffer injuries and diseases from agricultural operations caused by machines, animals, and chemicals. Thus, despite the role of ULF in warding off hunger and poverty in urban areas, it has hazards associated with it. Given that significant livestock activities are being carried out in the Southeast zone of Nigeria, it is necessary to assess urban farmers' knowledge of hazards associated with urban livestock farming in the Southeast zone of Nigeria. Specifically, the objectives of this study were to: (i) describe socioeconomic characteristics of urban livestock farmers; (ii) assess respondents' knowledge of hazards associated with urban livestock farming; and (iii) identify strategies to minimize these hazards.

## Hypotheses

(i) To test the influence of 12 socio-economic characteristics of urban livestock farmers on the farmers' knowledge of the hazards associated with ULF

(ii) To test farmers' correct knowledge about 11 selected hazards associated with ULF in Enugu, Imo, and Ebonyi states of Nigeria.

## Methodology

### Study Area

Nigeria is divided into six geopolitical zones namely, Northeast, Northwest, Northcentral, Southeast, Southwest, and Southsouth zones. The study was conducted in the southeast geopolitical zone of Nigeria. The southeast is made up of five states viz: Enugu, Anambra, Imo, Abia, and Ebonyi States. The area stretches from latitude 04°15′N to latitude 07°00′N and longitude 05°34′E to longitude 09°24′E (17).

The zone has so many urban towns with growing population. Such urban towns within the zone include: Enugu, Aba, Umuahia, Owerri, Awka, Orlu, Abakaliki, Okigwe, Onitsha, Nsukka, and Afikpo. Observations show that a lot of urban agricultural activities take place in these towns. Many crops are grown along roadsides, near refuse dumpsites and open spaces within the towns. Many of the urban households also keep farm animals including poultry, sheep/goats, and pig.

### Population and Sampling Procedure

The population for the study comprised all urban livestock farmers in the Southeast zone of Nigeria. Out of the five states that make up the zone, three were selected using simple random sampling technique. These states are Ebonyi, Enugu, and Imo States. Each state has three senatorial zones. In each state, two out of the three zones were selected through random sampling technique. In Ebonyi State: Ebonyi South and Ebonyi Central were selected while in Enugu State, Enugu North and Enugu Central were selected. In Imo State, Owerri and Orlu zones were selected, thus making a total of six zones.

In each zone, a major urban center was purposively selected making a total of six urban centers. Five urban (political) wards were purposively selected from each urban center based on their involvement in urban livestock farming (ULF), making a total of 30 urban wards. From each sampled ward, a list of urban farmers was drawn. Seven urban farming households were purposively selected based on their involvement in ULF; giving a sample size of 210 respondents. Heads of households were interviewed.

### Data Collection Method

Data were collected through interview schedule, focus group discussion (FGD) and observation. Three FGDs (comprising 8–10 members in each state) were conducted, one in each state. The instrument used for data collection was validated by three academic staff from the Department of Agricultural Extension, University of Nigeria, Nsukka to give their opinions on the relevance and adequacy of the instrument in accordance with the objectives of the study.

### Variables Specification

To assess farmers' knowledge of hazards associated with ULF, the respondents were required to provide answers to specific statements about hazards from livestock keeping. Against each specific statement, respondents were requested to tick “True” for a correct statement and “False” for an incorrect one. A correct response was scored one (1) while an incorrect one was scored zero (0). The knowledge index of each respondent was determined by adding up the scores for the knowledge statements. The knowledge indices of the respondents were used to run regression analysis. Furthermore, the total score for each statement was converted to percentage and a score of ≥80% was regarded as very high knowledge, 60–79% as high knowledge, 40–59% as moderate knowledge, 20–39% as low knowledge while ≤19% was regarded as very low knowledge [a modification of the classification of (18)].

To ascertain strategies to minimize the hazards associated with ULF, a list of possible strategies was presented to the farmers. They were expected to rate them on a three-point Likert-type scale with regard to how effective the strategies are in minimizing hazards associated with ULF. The scales were assigned values as follows: very effective = 2, effective = 1, and not effective = 0. A mean score of 1.0 was obtained. Any item with a mean of 1.0 and above was regarded as effective strategy to minimize hazards from urban farming while mean <1.0 was regarded as ineffective. The urban farmers were also requested to specify any other strategy not listed. The strategies specified were included in the list and scored for each respondent on a three point Likert type scale as explained above.

### Data Analysis

Data were presented in percentages and mean scores. Hypothesis 1 was tested using a multiple regression analysis. This is represented by the equation.

$$\begin{array}{l} \text{Y}={\text{b}}_{\text{o}}+{\text{b}}_{1}{\text{X}}_{1}+{\text{b}}_{2}{\text{X}}_{2}+{\text{b}}_{3}{\text{X}}_{3}+{\text{b}}_{4}{\text{X}}_{4}+{\text{b}}_{5}{\text{X}}_{5}+{\text{b}}_{6}{\text{X}}_{6}+\\ {\text{ b}}_{7}{\text{X}}_{7}+{\text{b}}_{8}{\text{X}}_{8}+{\text{b}}_{9}{\text{X}}_{9}+{\text{b}}_{10}{\text{X}}_{10}+{\text{b}}_{11}{\text{X}}_{11}+{\text{b}}_{12}{\text{X}}_{12}\;+\text{e} \end{array}$$

Where

Y = Knowledge score

B_o_ = Coefficient of the model

b_i_–b_12_ = Coefficient of the various socio-economic characteristics

X_1_ = Age (in years)

X_2_ = Marital status (single-1, married 0)

X_3_ = Educational level (number of years spent in school).

X_4_ = Membership of social organizations (1 if a member, 0 otherwise)

X_5_ = Sex (Male = 1, female = 0)

X_6_ = Extension contact (contact = 1, no contact = 0)

X_7_ = Urban livestock farming experience (years)

X_8_ = Household size (number of people eating in one pot)

X_9_ = Major occupation (civil service = 1 others = 0)

X_10_ = Stock size (total number of animals reared)

X_11_ = Years spent in the city (continuous)

X_12_ = Income from sale (annually)

e = error term.

Hypothesis 2 was analyzed using analysis of variance (ANOVA) to compare farmers' knowledge of hazards associated with ULF in the three states (Ebonyi, Imo, and Enugu). *Post-hoc* test was carried out using Duncan's Test. All analyses were done at 5% level of probability. The Statistical Product for Service and Solutions (SPSS) was used for the analysis.

## Results

### Socioeconomic Characteristics of Respondents

The mean age of the respondents was 49.1 years (Table 1). The majority (61.4%) of respondents was male and 88.2% were married. The mean years spent in school was 12.2 years while the average household size was six persons. About 47% of the respondents were migrants. The mean years spent in city was 21.65 years. The mean years of farming experience was 12.7. The majority (78.6%) of the respondents belonged to at least one social organization while 47.7% had access to credit and only 7.1% indicated farming as their main occupation.

**Table 1:** Socioeconomic characteristics of respondents.

**Socio-economic characteristics**	**%**	**Average**
**Age**		
20–29	1.9	
30–39	10.9	
40–49	40.0	49.1
50–59	31.0	
60–69	12.4	
70–79	2.4	
80 and above	1.4	
**Sex**		
Male	61.4	
Female	38.6	
**Marital status**		
Married	88.2	
Single	3.8	
Widowed	5.2	
Divorced	2.8	
**Educational level**		
No formal education	13.3	
Primary education	20.5	
Secondary education	38.5	
Tertiary education	19.1	
Above tertiary education	8.6	
Mean years spent in school		12.2
**Household size**		
1–5	36.2	
6–10	53.8	
11–15	3.3	6.0
>15	6.7	
**Migration status**		
Migrants	46.7	
Indigenes	53.3	
**Years spent in the city**		
1–10	23.3	
11–20	32.4	
21–30	18.6	21.65
31–40	10.5	
41–50	5.7	
>50	9.5	
**Urban livestock farming experience**		
1–10	56.2	
11–20	29.0	
21–30	9.0	12.7
31–40	2.9	
>40	2.9	
**Extension contact**		
Yes	70	
**Membership of social organization**		
Yes	78.6	
**Access to credit**		
Yes	47.7	
**Major occupation**		
Civil service	45.7	
Trading	16.2	
Politics	0.5	
Retiree/pensioner	25.8	
Artisan	4.7	

### Type of Animals Kept and Rearing System

Predominant animals reared by the urban dwellers were broiler chickens (78.1%), indigenous chicken (37.6%), goat/sheep (33.8%), layers (33.3%), and turkeys (30.5%) (Table 2).

**Table 2:** Type of animals kept.

**Type of animal**	**Enugu %** ***n* = 70**	**Imo %** ***n* = 70**	**Ebonyi %** ***n* = 70**	**All %** ***n* = 210**
Broilers	87.1	75.7	71.4	78.1
Layers	35.7	42.9	21.4	33.3
Turkeys	57.1	24.3	10.0	30.5
Indigenous chicken	31.4	37.1	44.3	37.6
Goat/sheep	20.0	25.7	55.7	33.8
Pig	22.9	10.0	10.0	14.3
Cattle	0.0	0.0	4.3	1.4
Duck	1.4	0.0	0.0	0.5
Rabbit	2.9	0.0	1.4	1.4

As shown in Table 3, the dominant rearing system for broilers (92.1%) and layers (100%) was intensive. All the pigs, cattle and rabbits were also under intensive management system. The majority (89.1%) of respondents kept turkey under intensive system. A greater proportion (58.2%) of the respondents engaged in extensive rearing system for indigenous chicken. Goats and sheep were mainly managed under semi-intensive (39.4%) and extensive systems (35.2%). About 25% of the respondents kept their goats/sheep in intensive system.

**Table 3:** Rearing system of respondents.

	**Intensive** ** (%)**	**Extensive** ** (%)**	**Semi-intensive** ** (%)**
Broilers (*n* = 164)	92.1	3.1	4.8
Layers (*n* = 75)	100.0	0.0	0.0
Turkeys (*n* = 64)	89.1	6.3	4.6
Local chicken (*n* = 79)	14.0	58.2	27.8
Goat/sheep (*n* = 71)	25.4	35.2	39.4
Pig (*n* = 30)	100.0	0.0	0.0
Cattle (*n* = 3)	100.0	0.0	0.0
Duck (*n* = 1)	0.0	100	0.0
Rabbit (*n* = 3)	100.0	0.0	0.0

### Disposal of Waste From Livestock

Most (97.1%) of the waste (dung) was used by the urban farmers for crop cultivation while a good number (60%) sold theirs and 12% gave out to neighbors and friends (Table 4). The dung (untreated) given out or sold was also used as manure for crop cultivation.

**Table 4:** Method of wastes disposal from livestock.

Waste disposal method^*^	**%**
Used for crop cultivation by respondents	97.1
Sold	60.0
Give out to neighbors and friends	12.4
Keep along road side or in refuse dump	4.9

* *Multiple response*.

### Knowledge of Hazards Associated With Urban Livestock Keeping

High knowledge of the hazards associated with urban livestock keeping was recorded with mean scores of 71.2, 68.0, and 65.3% for respondents in Enugu, Imo, and Ebonyi States, respectively (Table 5). For the three states combined, a mean score of 68.3% was recorded. Specifically, making environment dirty (87.2%), causing accidents in urban areas (83.8%), depositing animal dung on compound, dung breeding disease (91.6%), and dung causing bad odor (83.2%) were identified to be associated with urban livestock keeping by a high proportion of respondents. In-depth discussion with the farmers revealed that diseases like tuberculosis, worms and tetanus can be contacted through livestock keeping.

**Table 5:** Correct knowledge of hazard associated with urban livestock keeping.

**Knowledge item**	**Enugu** ** (%)**	**Imo** ** (%)**	**Ebonyi** ** (%)**	**All** ** (%)**
Livestock in urban areas can destroy crops	58.5	62.1	57.1	59.2
Keeping livestock in urban areas makes the environment dirty	93.8	81.0	85.7	87.2
Livestock can cause accidents in urban areas	84.6	89.7	76.8	83.8
Livestock can destroy fences and pipelines	63.1	69.0	50.0	60.9
Livestock in urban areas can deplete water sources	70.8	48.3	55.4	58.7
Animal product from intensive system can be contaminated with heavy metals	40.0	36.2	41.1	39.1
Diseases from livestock can affect human beings	60.0	55.2	66.1	60.3
Livestock farming can cause climate change	40.0	31.0	39.3	36.9
Animal dung in the compound is a breeding ground for disease causing vector	92.3	94.8	87.5	91.6
Waste from livestock has bad odor/smell	86.2	86.2	79.8	83.2
Animals in urban areas can make a lot of noise	93.8	94.8	82.1	90.5
Mean percentage scores	71.2	68.0	65.3	68.3

### Strategies to Minimize Hazard From Urban Livestock Keeping

As shown in Table 6, perceived effective strategies to minimize hazard from urban livestock keeping included proper disposal of waste ($\bar{x}$ = 1.53), cleaning animal house weekly ($\bar{x}$ = 1.52), restraining animals ($\bar{x}$ = 1.38) from entering farms and neighbor's residence. Others included seeking veterinary services ($\bar{x}$ = 1.47) to keep diseases at bay, feeding animals well ($\bar{x}$ = 1.31) to limit noise.

**Table 6:** Strategies to minimize hazards from urban livestock keeping.

**Strategies**	**Mean $\left(\bar{x}\right)$**	**SD**
Proper disposal of waste	1.53^*^	0.639
Use of waste from livestock for crop cultivation	1.40^*^	0.651
Reducing the number of animals	0.99	0.762
Restraining animals	1.38^*^	0.619
Feeding animals well	1.31^*^	0.786
Seek veterinary services	1.47^*^	0.604
Cleaning animal house weekly	1.53^*^	0.555
Keeping all animals in intensive system	0.81	0.556
Provision of vital information by extension services	1.53^*^	0.594

* *Effective strategies*.

### Factors Influencing Knowledge of Hazards Posed by Urban Livestock Keeping

The regression results in Table 7 shows that the socio-economic characteristics of the farmers have a significant (*F* = 6.366) influence on knowledge of hazards posed by livestock keeping. The *R* square value and the adjusted R square value were 0.303 and 0.256, respectively. Nearly 26% of the variance in the knowledge of hazard from livestock keeping was explained by the variables included in the model. Years of farming experience (t = −2.216; *P* = 0.028) and stock size (t = −2.347; *P* = 0.020) had significant negative influence on knowledge of hazards from livestock keeping. Membership of social organization (t = 2.512; *P* = 0.013) and number of extension contact (t = 3.503; *P* = 0.000) had significant positive influence on farmers' knowledge of hazards generated by keeping livestock in urban area.

**Table 7:** Factors influencing knowledge of hazards posed by urban livestock keeping.

**Variables**	**Unstandardized coefficient**		**Standardized coefficients**		
	**B**	**Std. Error**	**Beta**	**t**	***p*-value**
(Constant)	6.689	2.130		3.141	0.002
Age	−0.0014	0.031	−0.043	−0.454	0.650
Sex	−0.169	0.508	−0.025	−0.333	0.739
Marital status	1.525	0.850	0.134	1.796	0.075
Years spent in school	−0.123	0.066	−0.161	−1.860	0.065
Household size	0.062	0.122	0.44	0.508	0.612
Years spent in the city	0.005	0.023	0.022	0.232	0.817
Years of urban livestock farming experience	−0.093	0.042	−0.228	−2.216	0.028^*^
Membership of social organization	1.506	0.599	0.191	2.512	0.013^*^
Number of extension contact	1.910	0.502	0.293	3.803	0.000^*^
Stock size	−0.424	0.181	0.180	−2.347	0.020^*^
Major occupation	0.037	0.538	0.006	0.069	0.945
Estimated income in a year	2.422	0.000	0.111	1.556	0.122

*Dependable variable: Knowledge score R Square = 0.303; R^2^ = 0.256; F-value = 6.366; P ≤ 0.05*.

* *Significant*.

### Variation in Knowledge of Hazards Associated With Urban Livestock Farming in the Three States

There is no significant (*F* = 4.317; *P* = 0.015) variation in the knowledge of hazards associated with urban livestock keeping, among urban farmers in the three states. The mean of knowledge of urban farmers in Ebonyi State ($\bar{x}$ = 5.743) did not differ significantly with the mean of respondents in Imo State ($\bar{x}$ = 6.200). However, the mean for respondents in Enugu State ($\bar{x}$ = 7.271) differed significantly from that of respondents in Ebonyi and Imo States.

## Discussion

Broiler chickens were the most reared in the study area. This may be because the demand for broiler is higher than other animal types due to its comparative affordability (19). Secondly, they are capable of bringing faster returns than ruminants and pigs. These findings corroborate those of Njengbwen and Njengbwen (20) and Alarcon et al. (21) who reported poultry as the most popular animal production activity in Uyo urban area, Nigeria and Nairobi Kenya, respectively. It was also found that sheep and poultry are the most common reared animals in the urban areas of West Africa (22). However, this is contrary to the study of Yusuf et al. (23) who reported that majority of urban farmers in Katsina State, Nigeria reared sheep and goats. The most common livestock kept in Kisumu, Kenya according to Barnes et al. (24) were cattle followed by goats because they do not need a lot of care and do not present major health problems.

Goats and sheep were reared under extensive and semi intensive system of production. Also indigenous chickens were kept under extensive system. Animals kept under extensive system of production, scavenge for food, roamed the streets and could cause accidents on roads and water ways. In Nairobi Kenya, small scale farmers mainly fed their animals through scavenging while large scale farmers and fatteners grazed their animals in pastoral areas in the urban area (21). Discussions with the respondents revealed that during the rainy season, when crops are on the fields, most goat/sheep are kept under intensive system to avoid destruction of crops. Since most of the respondents were engaged in intensive management system, majority used feed supplements and veterinary drugs for their animals. Yusuf et al. (23) found out that most of the households in cities in West Africa provide supplemental feed to their animals. The most common feedstuffs used for supplementation included maize bran, cowpea and groundnut hay, cereal straws, fresh grass, brewer's spent grain, rice bran, and cassava peels. This could be linked to the affordability and availability of these feedstuffs, as majority of the items is plants by-products that are unfit for human consumption. Pig owners during FGD indicated that they collected wastes from restaurants and hotels to feed their pigs. This might have been cheaper for them than formulated feed. In Tamale, Ghana and Ouagadougou, Burkina Faso feeding pigs at home was common, but scavenged feeding was also practiced by 35.9% of pig keepers across the two cities (22). Cattle in the study area were kept under intensive system of production which is not a practice in Nigeria. This may be because they were in small numbers (some had just one or two) and equally most of the respondents were engaged in other income activities and do not have time to move the animals around to graze, since there is limited empty land in the urban areas.

The dung used for crop production was not treated before use as indicated during FGD. This practice contributes to contamination of the environment as well as foods with pathogenic and antimicrobial resistant bacteria. Oladipo et al. (25) in their study in Kwara State Nigeria noted that farmers deposited farm waste in nearby streams and rivers. This may have serious impact on health and general well-being of both humans and animals with its attendant serious economic implications. The use of manure on crop is risky due to microbial contamination of crops and vegetables. *Salmonella spp*. and *Escherichia coli* were reported to be the mirco-organisms identified from manure to contaminate crops in Niamey, Niger (26). The poor health care to livestock herds can be an additional risk to humans and animals through poor livestock waste disposal (uncovered manure heaps and slurry pits, livestock waste disposal in the streets) and also through direct contact of humans with the animals. According to Greentumble (27), 13 livestock-related diseases that can affect humans cause up to 2.4 billion cases of human illness. Makita et al. (28) noted that informal markets for food increases the spread of zoonotic diseases in cities, in urban and peri-urban areas of Kampala in Uganda. Ways to reduce nitrogen losses (which causes climate change) into the atmosphere include proper handling of livestock manure (e.g., covering manure heaps) and feeding management (feeding animals based on their nutrient requirements) (26). Farmers therefore need technical advice on pre-treatment of dung by composting to reduce transfer of pathogens. Generally, agriculture extension advice is necessary to provide new knowledge and ideas to farmers in order to bring change and improve the lives of farm families (29). Animal products may become contaminated by heavy metals if animals feed or drink water polluted by traffic (30, 31). Kabir et al. (32) noted that heavy metal contamination was evident in dairy farming system in Bangladeshi because of industrial effluents. This means that humans and animals can come into contact with toxic metals through direct ingestion, skin contact and even by inhalation (33); the continuous intake of such contaminated products may not only be detrimental for the affected individuals but also have a negative effect on the economy of the affected areas (34). For example, animals reproduce less due to contaminated feed (33, 35). Food safety is very necessary for humans to be healthy; to make sure food is safe, all possible avenues of contamination have to be checked along the food chain (36). Since most of the respondents engaged in intensive poultry production, it is necessary that they know these facts and take precautions. Gerber and Steinfed (37) noted that regulations are needed to deal with heavy metals and drug residues.

Knowledge of hazards associated with urban livestock keeping in the three states was considered high. A high proportion of respondents could identify that making environment dirty, causing accidents in urban areas, animal dungs in compound constituting sources of infectious agent and bad odor were associated with urban livestock. Although the knowledge was high, it didn't correspond to what they practiced as noticed during data collection and findings from FGDs. For example animal dung was littered in compounds and roadsides; waste water from production activities was not properly disposed off among others. Inappropriate management of animal waste gives room for infection through vectors such as insects, rodents, dogs, wild beasts, birds, and others (38). This can lead to zoonosis and spreading of diseases among animals. In developed countries, 20% of human illnesses are as a result of zoonotic diseases and the situation is undoubtedly worse in developing countries (12). Zoonotic diseases are of concern in developing countries and show a correlation with poverty, hunger, and livestock rearing (12). The government has to exercise some control of ULF by putting some measures in place (insist on intensive system of production) or use statutes already in place. The finding that livestock destroy crops and causes accident may be associated with free roaming animals like local chicken, sheep, and goats in the streets of towns. Armar-Klemesu and Maxwel (39) and Okantah et al. (40) indicated that among other environmental issues livestock reared in urban areas could roam and cause traffic accidents, destroy crops, ornamental plants, lawn, water pipes, and fences and this may cause conflict with neighbors. Urban farmers in Morogoro, Tanzania identified erosion, dirtiness, noise, accidents, destruction of gardens, and water sources as hazards posed by livestock keeping in the area (41). Similarly, in Nakuru, Kenya, farmers indicated bad smell, erosion, diseases, destruction of fences and flowers as hazards from livestock keeping (42).

A study in Nigeria found that ULFs suffer high losses from stealing and are more likely to report emotional stress and discouragement (43). This confirms that regulations of ULF and especially livestock rearing are weak and common risk management is not effective. These should be strengthened so that the benefits of ULF can be maximized. However, commands and regulations not properly handled can make things worse. A study in Kampala shows that dairy farmers who were more harassed by public authorities had fewer good practices (44). It is necessary to work with the farmers to put these regulations in place.

A high proportion of the respondents, however, did not know that animal products from intensive system can be contaminated with heavy metals and that livestock can contribute to climate change. Animal products (red meat, poultry meat, and eggs) may be contaminated with pesticides if kept in an intensive system (45). Animal products may also become contaminated by heavy metals if animals feed or drink water polluted by exhaust fumes from automobiles in cities (45). Without appropriate handling and control of heavy metals, they may not only be a threat to animal health and a risk of heavy losses of livestock but also a threat to human health (46). This may invariably cause health implications to humans who consume them. Since most of the respondents engage in intensive poultry production, it is necessary that they know these facts and take precautions.

Respondents did not know that livestock farming can contribute to climate change. The contribution of livestock farming to climate change has been well-established (47). Grossi et al. (48) opined that livestock production systems globally contribute to human-induced green house gases, the cause of global warming. This is likely to increase in the future because demand for livestock product is rising rapidly due to increasing urbanization (49). Intensification of livestock farming can increase methane emission and other green house gases per unit weight of livestock produced. It is therefore necessary to create awareness, so that farmers know that methane and nitrous oxide are gases which have effect on global warming and these gases arise from manure storage and the use of organic/inorganic fertilizers.

The findings of this study therefore suggest that ULF can pose some threats to health and environment. Urban authorities have not accepted farming as a formal urban land use because of perceived health and environmental risks. However, prohibitive laws have proved to be largely ineffective. Hence, policies are required that lead to an active management of the potential health and environmental risks associated with ULF. Government should ensure healthy production systems in order to reduce hazards which are of concern to human and animal health. Health and safety of the farmer and that of his staff, along with an environment that is safe, are preconditions of having an effective farm venture.

Reducing the number of animals and keeping animals under intensive system were considered as strategies to minimize hazards. Reducing the number of animals may have a negative effect on the producers, because it may reduce their income. On the other hand, intensive system of production may lead to increased profitability of each animal and consequently increase income if the fewer number are well-managed with good husbandry practices as advised by the extension workers. Intensive system of production will also prevent crop destruction and accidents in urban centers.

The negative influence of farming experience and stock size on knowledge of hazards obtained in this study may be attributed to the fact that farmers who have been keeping livestock for many years or who have large stock size may be more interested in the benefits they derive from livestock keeping than knowing and paying attention to the hazards associated with the venture. This is dangerous, as they may not make any efforts to see that hazards are reduced. It is necessary to draw their attention to this so that they become aware and take precautions to reduce hazards which are detrimental to human health. It can also be that farmers with high farming experience are “old school” and hence think they know it all, and are reluctant to call for extension advice. The positive influence of membership of social organization and extension visits on knowledge of hazards generated by keeping livestock in urban areas may be attributed to the fact that belonging to an association gives farmers opportunity to get information from their contacts. Farmers working closely and cooperatively may share knowledge and information on hazards with one another and with other communities. There is power in networking as this may encourage knowledge sharing and may lead to enhancement and sustainability of ULF. The more the number of extension visits, the higher the chances that the farmers obtain information on hazards posed by keeping livestock in urban areas and therefore the more knowledgeable they become with respect to the hazards. Extension service is one of critical components of agricultural development. It contributes to the reduction of hunger and poverty by improving knowledge and information sharing among farmers. This may increase farmers' capacity which may go a long way to increase profits and improve food security. However, it is derived from literature that extension does not reach out to urban farmers as much as they do to rural farmers. There is a believe that agriculture takes place only in the rural areas and so majority of urban farmers are deprived of sufficient and suitable agricultural information and extension visits as shown on Table 1. There is an urgent need to make improved access to information that is adequate and relevant for urban farmers by increasing the number of extension visits.

The significant variation in the knowledge of hazards associated with urban livestock keeping among urban farmers in the three states, imply that individual farmer's extent of knowledge of hazards was higher in Enugu State compared with Imo and Ebonyi States. A deliberate regional policy to educate farmers on knowledge of ULF in Southeast Nigeria should therefore commence in Imo and Ebonyi States.

## Conclusion

Farmer had a high knowledge of hazards caused by livestock keeping in urban areas. However, observations showed that what they know is contrary to actual practice in their farms. They therefore need expert advice to enable them marry knowledge with practice as this will reduce hazards of ULF. Farmers did not know that rearing of livestock causes climate change and that heavy metals can contaminate livestock products. Also they didn't know that pretreatment of manure is necessary before applying to crops in order to reduce contamination with microorganisms and to reduce nitrogen gas emission into the atmosphere. One way to increase awareness and knowledge could be by comprehensive campaigns in urban areas providing educational and illustrative information and participatory practical training courses. More importantly, farmers must trust their educators, and training must be performed with respect to the beliefs and norms of the region. The study also highlights that the extension contact is low, and that there is a significant positive relationship between extension contact and knowledge of hazards. The information that extension visit rural farmers more and invariably give more information to them than urban farmers should be reconsidered by agricultural extension organizations. There is an urgent need to make improved access to information that is adequate and relevant for urban farmers by increasing the number of extension visits.

## Data Availability Statement

The raw data supporting the conclusions of this article will be made available by the authors, without undue reservation.

## Author Contributions

AA collected the data. JC wrote up the article. CA did the analysis while EI designed and proof read the study. All authors contributed to the article and approved the submitted version.

## Conflict of Interest

The authors declare that the research was conducted in the absence of any commercial or financial relationships that could be construed as a potential conflict of interest.
